# Psychometric evaluation of the MEDication Literacy Assessment in Geriatric Patients and Informal Caregivers (MED-fLAG) instrument using Rasch analyses in a sample of hospitalised older adults

**DOI:** 10.1016/j.ijnsa.2026.100610

**Published:** 2026-06-24

**Authors:** Jenny Gentizon, Laura Mortelmans, Nadège Tapsoba, Nathalie Rebetez, Anne-Lore Bynens, Carole Schol, Louise Vanheusden, Anneleen Van De Weyer, Roger Hilfiker, Tinne Dilles, Cedric Mabire

**Affiliations:** a Institute of Higher Education and Research in Healthcare, Lausanne University Hospital and University of Lausanne, Switzerland; b Department of Nursing Science and Midwifery, Centre for Research and Innovation in Care (CRIC), Nurse and Pharmaceutical Care (NuPhaC), Faculty of Medicine and Health Sciences, University of Antwerp, Antwerp, Belgium

**Keywords:** Medication literacy, Patient-reported outcome measures (PROMs), Older adults, Rasch analyses, Psychometrics, Scale development

## Abstract

**Background:**

Medication literacy is critical for older adults managing complex medication regimens, yet few measures have been developed specifically for this population. The MEDication Literacy Assessment of Geriatric patients and informal caregivers (MED-fLAG) was developed as a multidimensional self-reported measure for older adults and informal caregivers. The instrument uniquely captures skills related to both prescribed and non-prescribed medications, including herbal and nutritional supplements. Although the MED-fLAG previously demonstrated satisfactory content validity, further evaluation of its psychometric properties was warranted.

**Objectives:**

To evaluate the psychometric properties of the MED-fLAG using Rasch analyses.

**Methods:**

A cross-sectional study was conducted among French- and Dutch-speaking hospitalized older adults in Switzerland, Belgium, and the Netherlands. Hospitalized participants aged ≥ 65 years who had managed their medications for at least three months were included. Rasch modelling using the Partial Credit Model was applied to assess unidimensionality, item fit (goodness-of-fit), item hierarchy (item difficulty calibration), person separation reliability, and differential item functioning across language groups.

**Results:**

Among the 582 respondents, the mean age was 73.9 years, with a standard deviation of 11.4 years. Analyses were conducted on 563 responses, including 356 from the Dutch-speaking sample and 207 from the French-speaking sample. Rasch analyses supported the measurement properties of the Functional subscale (with 19 items) and Interactive subscale (with 13 items), including satisfactory unidimensionality, item fit, and reliability. However, the Critical subscale (with 21 items) displayed signs of multidimensionality and substantial differential item functioning across languages. Person separation indices fell below the recommended threshold, indicating moderate discrimination, and Wright maps highlighted the need for more challenging Interactive items and easier Critical items.

**Conclusions:**

These findings support the MED-fLAG as a promising instrument while underscoring the need for further refinement, particularly within the Critical medication literacy subscale. In the future, this standardized measure may support the formal assessment of medication literacy among older adults and informal caregivers to ensure that medication regimens align with patients’ abilities. In the context of early hospital discharge, the MED-fLAG may provide valuable information to guide individualized care planning and help prevent medication-related problems. Future research should also explore the development of an optimized and potentially shorter version of the MED-fLAG to enhance feasibility in routine clinical practice. Further psychometric evaluation of a revised version of the MED-fLAG is needed before the instrument can be confidently recommended for research and clinical use

**What is already known about the topic?**

• Managing medications in older adults is a complex self-care activity that relies on multiple cognitive and social skills, conceptualised as medication literacy.
• Most existing medication literacy measures focused primarily on functional skills, overlooking the interactive and critical dimensions.
• Nurses play a central role in supporting older adults’ medication literacy but currently lack validated measures to systematically assess patients and tailor interventions accordingly.

Alt-text: Unlabelled box dummy alt text

**What this paper adds**

• The MEDication Literacy Assessment in Geriatric patients and informal caregivers (MED-fLAG) is the first geriatric-specific, multidimensional measure of medication literacy evaluated using Rasch analyses.
• Rasch analyses indicated satisfactory psychometric performance of the Functional and Interactive medication literacy domains while highlighting areas for refinement in the Critical medication literacy domain.
• This study also provided detailed insights into item functioning, targeting, and cross-language equivalence of the MED-fLAG, informing future refinement and validation studies.

Alt-text: Unlabelled box dummy alt text

## 1 Introduction

Older adults (≥ 65 years) frequently manage complex medication regimens that combine prescribed and non-prescribed medications. These regimens often involve multiple dosages, formulations, and administration schedules, making medication management particularly challenging (Maidment et al., 2020). Medication self-management is therefore a demanding self-care activity (Mickelson et al., 2016) that requires a range of cognitive and social skills, collectively referred to as *medication literacy* (Pouliot et al., 2018, King et al., 2011).

Medication literacy is a multidimensional concept that encompasses the knowledge and skills to obtain, understand, communicate, calculate, and apply medication-related information for informed decision-making (Gentizon et al., 2022a, Pantuzza et al., 2020). In the geriatric population, medication literacy has recently been defined as the degree to which older adults and/or their informal caregivers can develop and maintain functional, interactive, and critical medication literacy. Functional skills involve understanding, preparing, and self-administering medications, as well as monitoring their effects. Interactive skills reflect the ability to engage with healthcare providers, express concerns and preferences, and participate in medication-related decisions. Critical skills enable older adults to assess the relevance and quality of information, set up strategies to integrate medication taking in a daily routine and keep the control over their medication even when the situation changes (prescription changes, occurrence of medication-related problems) (Gentizon et al., 2022a). Adequate medication literacy is therefore essential to promote adherence, ensure safe use of medicines, and prevent harm (Chesser et al., 2016, Parekh et al., 2018). Reflecting its clinical and public health significance, the World Health Organization has identified medication literacy promotion as a global priority for medication safety, especially in contexts of polypharmacy, high-risk medications, and transitions of care (Sheikh et al., 2019, World Health Organization, 2017).

Nurses play an important role in supporting and developing older adults’ abilities to self-manage their own medications safely and effectively (Advinha et al., 2017). They are in a strategic position to identify patients' difficulties with their medications, to share this information with the interprofessional team (Dilles et al., 2021), to recommend simplification or de-prescribing of medication regimens where possible (Smith et al., 2019), and to plan for appropriate transitional care (Mardani et al., 2020, Tobiano et al., 2019). A formal assessment of medication literacy is the essential first step toward individualizing care plans and reducing medication-related problems. However, efforts to evaluate medication literacy are hindered by the lack of standardised measures tailored to older adults (Gentizon et al., 2021).

To address this gap, a new measure—the MEDication Literacy Assessment in Geriatric patients and informal caregivers (MED-fLAG)—was developed (Gentizon et al., 2021, 2022a). Originally created in French, this multidimensional measure is tailored to both older adults and informal caregivers, when they assume responsibility for medication preparation and administration – and uniquely covers prescribed as well as non-prescribed medications, including herbal and nutritional supplements, which are common among older adults but often overlooked in clinical assessments (Abolhassani et al., 2017, Shreffler-Grant et al., 2013, Advinha et al., 2017). The conceptualization of the instrument involved content validation by healthcare professionals, older adults, and informal caregivers (Gentizon et al., 2022b). Preliminary findings indicate that the MED-fLAG is a promising instrument for assessing medication literacy and guiding tailored interventions in geriatric care (Mortelmans et al., 2024). However, its psychometric properties require further evaluation.

The Item Response Theory offers advantages over classical test theory by modelling the probability of a response as a function of both a person’s ability and item difficulty, rather than assuming equal item contribution (Bond and Fox, 2013). Within this framework, Rasch analyses have become particularly valuable for evaluating measures, providing insights into unidimensionality, targeting, item bias, and internal consistency (Nguyen et al., 2014, 2015, Richtering et al., 2017). Previous studies demonstrated that Rasch analyses helped refine health literacy measures by detecting measurement gaps, item bias, and dimensionality issues, thereby strengthening validity and reliability (Farlie et al., 2021, Guttersrud et al., 2015). This method has already been applied to instruments such as the Health Literacy Questionnaire (Bessing et al., 2022), the eHealth Literacy Scale (Richtering et al., 2017), and the Korean Health Literacy Scale (Lee et al., 2009). However, only one medication literacy measure—the MedLitRxSE—has been evaluated with Rasch analyses (Sauceda et al., 2012).

The aim of this study was to evaluate the psychometric properties of the MEDication Literacy Assessment in Geriatric patients and informal caregivers using Rasch analyses. It focused on the following key aspects: unidimensionality, item fit (goodness-of-fit), item hierarchy (item difficulty calibration), person separation reliability, and differential item functioning (DIF).

## 2 Methods

### 2.1 Design

We conducted a cross-sectional study to evaluate the measurement properties of the MEDication Literacy Assessment in Geriatric patients and informal caregivers using the Partial Credit Model, an extension of the Rasch model for polytomous items (Fox, 1999, Masters, 2016). Reporting followed COnsensus-based Standards for the selection of health Measurement Instruments (COSMIN) standards for patient-reported outcome measures (Prinsen et al., 2018, Gagnier et al., 2021) and recommendations specific to Rasch analyses (Stolt et al., 2022, Linacre, 2011).

### 2.2 Population and settings

We recruited older adults prior to hospital discharge in Switzerland, Belgium (Flanders), and the Netherlands, enabling a cross-linguistic evaluation of the French and Dutch versions. For polytomous data, sample sizes of 250–500 are generally adequate (Hagell and Westergren, 2016). Since the MEDication Literacy Assessment in Geriatric patients and informal caregivers includes 56 items, recommendations for studies evaluating measurement properties suggest a minimum of seven participants per item, resulting in a required sample size of at least 390 participants (Prinsen et al., 2018).

Recruitment occurred in 22 clinical wards across five hospitals between 2022 and 2024. Convenience sampling was used, based on the following eligibility criteria: age ≥ 65; ≥ 3 months of medication self-management; prescription of ≥ 1 chronic medication (with or without over the counter or herbal products); personal responsibility for preparation and administration; and sufficient proficiency in French or Dutch. In Dutch sites, an additional criterion was polypharmacy (≥ 5 chronic medications daily). Exclusion criteria were age < 65 years; severe illness, end-of-life condition, or cognitive impairment, all judged clinically by the care teams; less than 3 months of medication self-management; or prescriptions involving short-term medication use only.

### 2.3 Study instrument

The MEDication Literacy Assessment of Geriatric patients and informal caregivers is a 56-item patient-reported outcome measure, originally developed in French, to assess functional, interactive, and critical medication literacy (Gentizon et al., 2022b) (Appendix A). Items are scored on 4-point ordinal scales reflecting either difficulty (3= not difficult at all to 0= very difficult/impossible) or frequency (3= always to 0= never), with an additional “does not apply to me” option. Higher scores reflect higher levels of medication literacy. The MED-fLAG subscales are summarized in Table 1.

**Table 1:** Description of the MED-fLAG subscales and associated items.

Subscales	Description	Items
Functional medication literacy [FML]	(…) is the degree to which patients or their informal caregivers: a) have a basic knowledge about the medications; b) know the purpose; c) understand the instructions related to the preparation and administration; d) are able prepare correct dosages (i.e., calculating); and e) are able to monitor of the effects and observe precautions.	22 items
Interactive medication literacy [IML]	(…) is the degree to which individuals: a) ask for clarification and understand explanations given by healthcare professionals; b) provide medication-related information; and c) inform about medication-related difficulties and problems.	13 items
Critical medication literacy [CML]	(…) is the degree to which individuals: a) seek reliable medication-related information; b) set up strategies and practical means to integrate medication taking in a daily routine; and c) have the control over their medication and adjust when the situation changes.	21 items

### 2.4 Translation procedures

Prior to this study, the MEDication Literacy Assessment in Geriatric patients was translated from French into Dutch following established guidelines for the translation and cross-cultural adaptation of patient-reported outcome measures (Acquadro et al., 2008, Beaton et al., 2000). A forward–backward translation procedure was conducted by professional translators at the University of Antwerp, fluent in both French and Dutch and experienced in healthcare terminology. One translator produced the forward translation into Dutch, and a second bilingual translator, blinded to the original version, performed the backward translation into French. The original and back-translated versions were compared to identify discrepancies and ensure conceptual and cultural equivalence. Any inconsistencies were resolved through consensus between the translators and the research team. The finalized Dutch version was then prepared for use in the study. However, no formal cognitive debriefing with Dutch-speaking patients was conducted after translation, which is acknowledged as a limitation, as pretesting could have further ensured semantic clarity and cultural relevance.

### 2.5 Characteristics of participants

Participant characteristics included age, sex, highest level of completed education, categorized according to the European Qualifications Framework as 1–4 (elementary and upper secondary education), 3–5 (federal diplomas and vocational training), and 6–8 (tertiary education, including academic bachelor, master, and doctoral degrees). Additional variables included living arrangement (living alone vs. not) and the number of self-managed medications, further classified to identify polypharmacy—defined as the daily use of five or more medications (Masnoon et al., 2017).

In the Swiss French-speaking sample, additional data were collected on self-rated health (very good, good, average, poor, or very poor) and functional status, assessed through self-reported difficulties in basic activities of daily living (dressing, bathing, eating, transferring, toileting) and instrumental activities of daily living (medication management, shopping, cooking, cleaning, laundry, transportation). For both basic activities and instrumental activities, responses were categorised as no difficulty, difficulty without help, or requiring help. In the Dutch-speaking sample (Belgium and the Netherlands), participants were asked specifically about difficulties with medication self-management, recorded as either without help or requiring help.

### 2.6 Study procedures

The investigators approached eligible patients within five days prior to discharge. Ward nurses pre-screened potential participants. A member of the research team then assessed eligibility, and subsequently presented study information, obtained informed consent, and conducted data collection. Participants completed the questionnaire either on paper or via a private REDCap link (Harris et al., 2019), with assistance from relatives permitted. Participants could complete the questionnaire across multiple sessions if needed. Paper responses were entered into REDCap (Harris et al., 2019) by the research team.

### 2.7 Data analyses

We conducted Rasch analyses for each subscale to assess unidimensionality, item fit, item hierarchy, person separation reliability, and differential item functioning by language. A summary of the measurement properties, their purpose, assessment methods, and thresholds is presented in Table 2.

**Table 2:** Summary of measurement properties, purpose, assessment methods and thresholds used.

Measurement property	Purpose	Assessment method	Thresholds
Unidimensionality (Kline, 2005; Linacre, 2011)	To determine whether each MED-fLAG dimension (functional, interactive, and critical) measures a single latent trait.	Principal components analysis of residuals (PCAR)	≥ 40% variance explained by first component; < 5% by second component; eigenvalue of first residual contrast < 2.0
Internal scale validity (Goodness-of-fit) (Bond & Fox, 2013; Linacre & Wright, 2000; Smith et al., 1998)	To assess how well each item fits the underlying construct of medication literacy.	Infit mean square statistics for each item	Acceptable range: 0.7–1.3
Hierarchy of the items (item difficulty calibration) (Boone et al., 2013; Linacre & Wright, 2000; Prieto et al., 2003)	To determine the ordering of items across the spectrum of medication literacy levels.	Item Separation Index (ISI), reliability coefficient (ISR), and Wright map	ISI ≥ 2.0 and reliability ≥ 0.7 (ISR) indicate adequate item calibration. Wright map should show good overlap between item difficulty and person ability
Person separation reliability (discrimination) (Bond & Fox, 2013; Linacre & Wright, 2000; Wright, 1996)	To evaluate the scale’s ability to differentiate among individuals with varying levels of medication literacy.	Person Separation Index (PSI) and reliability coefficient (PSR)	PSI ≥ 2.0 and reliability ≥ 0.7 (PSR) indicate adequate discrimination.
Differential Item Functioning (DIF) (Bond & Fox, 2013)	To examine whether items function equivalently across subgroups (here: language/country).	Rasch Welch t-test	*t*-values within the range of ±2 and DIF contrasts < 0.5 logits, indicate negligible DIF across subgroups

***Unidimensionality***—the basic assumption of Rasch analyses—implies that each scale measures a single construct (Kline, 2005, Stolt et al., 2022). We assessed unidimensionality for each MED-fLAG dimension. (Functional, Interactive, and Critical) using a principal component analysis of residuals. Criteria for adequate fit were set at ≥ 40% of variance explained by the first component, < 5% by the second, and an eigenvalue of the first residual contrast < 2.0 (Kline, 2005, Linacre, 2011).

***Internal scale validity*** was evaluated using item fit statistics, which assess how well each item corresponds to the underlying construct of medication literacy. Infit mean-square values between 0.7 and 1.3 were considered acceptable (Bond and Fox, 2013, Linacre, 2011, Smith et al., 1998). Good item fit indicates that the items contribute meaningfully to measuring the same underlying construct, supporting the internal validity of the scale. Conversely, items demonstrating misfit may reduce the instrument’s precision and should therefore be considered for removal, revision, or rewriting.

***Item hierarchy and calibration*** were explored through the Item Separation Index, the reliability coefficient, and Wright maps illustrating the distribution of item difficulty across medication literacy levels. Adequate calibration was defined as Item Separation Index ≥ 2.0 and reliability coefficient ≥ 0.7 (Boone et al., 2013, Prieto et al., 2003). The Item Separation Index and reliability coefficient offer indicators of the stability and reproducibility of the item hierarchy, while Wright maps offer a complementary visual representation of how well item difficulty is targeted to participants’ ability levels. Well-calibrated and appropriately targeted items indicate that the scale covers a suitable range of difficulty across the medication literacy continuum. This distribution improves the instrument’s ability to distinguish between different levels of medication literacy and enhances measurement precision. When The Item Separation Index or reliability coefficient values fall below recommended thresholds or Wright maps reveal poor targeting, such findings suggest the need to refine or add items to better represent the continuum of medication literacy and to improve measurement precision.

***Person separation reliability and the Person Separation Index*** were used to assess the instrument’s ability to distinguish between individuals with different levels of medication literacy. The person separation index ≥ 2.0 and reliability coefficient ≥ 0.7 were considered satisfactory (Bond and Fox, 2013, Wright, 1996). A Person Separation Index of 2 indicates that the scale can distinguish at least two strata (e.g. low vs. high medication literacy), with higher values reflecting better discrimination. High person separation index and reliability coefficient values indicate that the MED-fLAG can reliably differentiate respondents according to their medication literacy levels, supporting its discriminative validity. Conversely, lower values imply limited ability of the scale to distinguish among respondents, which can result either from suboptimal item targeting or from insufficient variability in the sample. In such cases, adjustments may involve refining or expanding the item set or ensuring greater heterogeneity in future samples to improve coverage of the construct and measurement precision.

***Differential item functioning*** was examined to test whether items functioned equivalently across language groups (French vs. Dutch). The Rasch Welch t-test was applied, with t-values within ±2 and Differential item functioning contrasts <0.5 logits indicating negligible differential item functioning (Bond and Fox, 2013). A minimum of 200 participants per group was considered necessary to detect meaningful DIF (Prinsen et al., 2018). The absence of differential item functioning supports cross-language equivalence and indicates that items are interpreted similarly across linguistic groups, strengthening the scale’s measurement invariance. Conversely, significant differential item functioning suggests that respondents with comparable levels of medication literacy perform differently depending on their language group, reflecting possible linguistic or cultural bias. Identifying such items is essential to determine whether translation review, cultural adaptation, or item revision is required to maintain equivalence across populations.

According to COnsensus-based Standards for the selection of health Measurement Instruments (COSMIN), interpretability parameters are not psychometric properties but offer complementary information that supports understanding and meaningful use of the scores. Interpretability was assessed by examining score distributions (mean and standard deviation or median and interquartile range). Floor and ceiling effects—defined as the proportion of respondents achieving the lowest or highest possible scores—were considered present when >15% of participants reached these extremes (De Vet et al., 2011). Assessing these effects helps determine whether the scale captures the full range of medication literacy and can sensitively detect differences or changes across respondents. Scores for each MED-fLAG dimension—Functional, Interactive, and Critical Medication Literacy—were reported as normalised z-scores on a 100-point scale.

Finally, we used descriptive statistics to summarise participant characteristics. Means and standard deviations were calculated for continuous variables (e.g., age, number of medications), whereas categorical variables (e.g., sex, education level, living arrangement) were described using absolute and relative frequencies. Group differences were tested using chi-square tests for categorical variables and independent t-tests for continuous variables. Analyses were conducted using Stata 17 (StataCorp, 2021), WINSTEPS (Linacre and Wright, 2000), and the TAM package in R (Robitzsch et al., 2020). Responses of “does not apply” were treated as missing, and participants with more than 50% missing MED-fLAG data were excluded from the analysis.

### 2.8 Ethics

The study was approved by ethics committees in Switzerland (CERVD 202301228) Belgium (ECOG099 and B3002022000169), and the Netherlands (I2021075 and METC20212023). All procedures adhered to the Declaration of Helsinki.

## 3 Results

### 3.1 Characteristics of the study participants

Among the 582 respondents, the mean age was 73.9 years, and 46.9% were female. French-speaking participants were significantly older than Dutch-speaking participants, and a greater proportion of the latter held academic-level (European qualifications 6–8) degrees. Polypharmacy (≥ 5 daily medications) was common overall (84.5%) and, as expected given the Dutch-specific inclusion criterion, nearly universal in the Dutch-speaking group. Living alone was more frequent among French-speaking participants. In addition, 64.8% of Dutch-speaking participants reported difficulties with medication self-management, while in the French-speaking subgroup, substantial proportions reported poor self-rated health and difficulties with basic and instrumental activities of daily living. Detailed characteristics by language group are shown in Table 3.

**Table 3:** Characteristics of the study participants.

	All (N= 582)	Dutch speaking (n= 365)	French-speaking (n= 217)	*p* value
Sex n (%)				
Female	273 (46.9)	151 (41.4)	122 (56.2)	0.0007 a
Age *M* (SD)	73.9 (11.4)	72.1 (12.3)	77.3 (7.2)	< 0.00001 b
Level of Education n (%)				
European Qualifications 1-4 (elementary school)	134 (23.0)	67 (18.4)	67 (30.8)	
European Qualifications 3-5 (vocational training)	232 (39.9)	135 (37.0)	97 (44.7)	
European Qualifications 6-8 (academic degrees)	209 (35.9)	158 (43.3)	51 (23.5)	
Not reported	7 (1.2)	5 (1.4)	2 (0.9)	
European Qualifications ≤ 3-5	366 (62.9)	202 (55.4)	164 (75.6)	< 0.00001 a
Living Situation n (%)				
Living Alone (yes)	233 (40.0)	117 (32.1)	116 (53.5)	< 0.00001 a
Not reported	1 (0.2)	1 (0.3)	0 (0)	
Self-rated health n (%)				
Poor or very poor	-	-	90 (41.5)	*NA*^c^
Basic activities of daily living (BADL)^d^				
Had difficulties (with/without help)	-	-	48 (22.1)	*NA*^c^
Instrumental activities of daily living (IADL)^e^				
Had difficulties (with/without help)	-	-	69 (31.8)	*NA*^c^
Medication self-management n (%)				
Had difficulties (with/without help)	-	243 (64.8)	-	*NA*^c^
Total number of medicines per day n (%)				
< 5 medicines	66 (11.3)	3 (0.8)	63 (29.0)	
5 – 10 medicines	425 (73.0)	311 (85.2)	114 (52.5)	
> 10 medicines	67 (11.5)	36 (9.9)	31 (14.3)	
Don’t know	2 (0.3)	2 (0.5)	0 (0)	
Not reported	22 (3.8)	13 (3.6)	9 (4.1)	
Polypharmacy (≥ 5 medicines)	492 (84.5)	347 (95.1)	145 (66.8)	< 0.00001 a

Note.

M= mean; SD= standard deviation

a χ² test

b t-Test

c Not applicable for group comparisons: as part of the functional status assessment, BADL and IADL were only collected in the French-speaking sample, while self-reported difficulties with medication self-management were assessed exclusively in the Dutch-speaking sample.

d BADL: dressing, bathing, eating, getting in/out of bed or an armchair and using the toilet

e IADL: medication self-management, shopping, cooking, cleaning, laundry, transportation

### 3.2 Psychometric evaluation of the MED-fLAG

Due to incomplete responses on the MED-fLAG (> 50% of missing data), we excluded 19 participants from the psychometric analyses. We conducted analyses on 563 participant responses, 356 in the Dutch speaking sample and 207 in the French speaking sample. Data from both language groups (French and Dutch) were pooled to optimise calibration precision, with differential item functioning analyses explicitly testing cross-language equivalence. This pragmatic approach improves parameter stability. However, pooled calibration may introduce bias in item difficulty estimates when differential item functioning is present. Therefore, results—particularly for the Critical subscale where substantial DIF was observed—should be interpreted with caution. The detailed differential item functioning plots presented in Appendix D identify the specific items responsible for these cross-linguistic differences and point out which items should be prioritised in future cross-language invariance testing. Detailed psychometric findings are presented below and summarized in Table 4.

**Table 4:** Psychometric properties of the MED-fLAG in Dutch-speaking and French-speaking sample (N= 563).

		Overall (N= 563)		
	Thresholds	FML (22 items)	IML (13 items)	CML (21 items)
Unidimensionality				
Explained variance of the first component	≥ 40%	47.2 (+)	59 (+)	41.5 (+)
Eigenvalue of first residual contrast	< 2.0	2.26 (±)	2.36 (±)	3.8 (±)
		FML (19 items)	IML (13 items)	CML (21 items)
Item fit (Goodness of fit)				
Infit mean square	0.7 - 1.3	0.73-1.34 (+) a	0.8 - 1.17 (+)	0.72-1.16 (+)
Hierarchy of items (difficulty calibration)				
Item-separation index (ISI) and reliability coefficient (ISR)	ISI ≥ 2.0 (≥ 0.7)	7.20 (0.98) (+)	5.08 (0.96) (+)	13.14 (0.99) (+)
Wright map inspection	Good alignment between item difficulty and person ability	Good alignment (+)	Good alignment (+)	Poor alignment (-)
Person-separation reliability (discrimination)				
Person-separation index (PSI) and reliability coefficient (PSR)	PSI ≥ 2 (≥ 0.7)	1.8 (0.76) (±)	1.53 (0.70) (±)	1.87 (0.78) (±)
Differential item functioning (DIF)				
DIF across subgroups, (language)	Negligible DIF if: *t*-values within the range of ±2 and DIF contrasts < 0.5 logits	Significant DIF in 2 items: FML1, FML10	Significant DIF in 4 items: IML28, IML30, ML25, IML33	Significant DIF in 14 items: CML40, CML41, CML42, CML43, CML46, CML47, CML48, CML49, CML50, CML51, CML52, CML54, CML55, CML56
Scores of the MED-fLAG				
MED-fLAG scores, by subscale Normalized z-scores on a 100-point scale	M (SD)	77.13 (22.2)	77.17 (20.7)	52.43 (14.8)
	Mdn (IQR)	84.2 (28.1)	85.4 (29.3)	53.2 (20.6)
Ceiling effect, % (n)	< 15 %	12% (70) (+)	0 (0) (+)	0 (0) (+)
Floor effect, % (n)	< 15 %	0.17% (1) (+)	0 (0) (+)	0 (0) (+)

Note.

(+)= satisfactory; (±) somewhat satisfactory; (-) unsatisfactory

M= mean; SD= standard deviation

Mdn= Median; IQR= Interquartile Range

a Item fit after removing three items in the Functional medication literacy subscale: FML2 and FML13 exhibited overfit (infit < 0: redundancy or overly predictable responses), while FML18 showed underfit (infit > 1.3: erratic or noisy responses).

### 3.3 Unidimensionality

Evidence of unidimensionality was generally supported for all three subscales, as the variance explained by the Partial Credit Model exceeded the recommended threshold of 40% (FML: 47.2%; IML: 59.0%; CML: 41.5%). However, the eigenvalues of the first residual contrast were slightly above the cutoff of 2.0 for the Functional (2.26) and Interactive (2.36) subscales, and substantially higher for the Critical subscale (3.8), indicating the presence of a secondary dimension. While the primary measurement construct appeared to be preserved, these residual patterns—particularly within the Critical Medication Literacy dimension—underscore the need for conceptual and item-format refinements.

### 3.4 Internal scale validity (item fit)

Item fit statistics indicated an overall good fit to the Partial Credit Model across the three MED-fLAG subscales. Within the Functional domain, analyses were first conducted on the original 22 items, after which three items were removed for misfit, consistent with recommended thresholds (acceptable infit range: 0.7–1.3). Specifically, FML2 and FML13 demonstrated overfit (infit < 0.7, suggesting redundancy or overly predictable responses), whereas FML18 demonstrated underfit (infit > 1.3, indicating erratic or noisy responses). Infit statistics were then recalculated for the remaining 19 Functional items. The removed items were (free translation from French): [FML2] *...list the names of the natural remedies you manage, e.g. herbal treatments, homeopathy, dietary supplements (by heart or with a support such as a prescription list);* [FML13] *...say how many times a day each medicine should be taken*; [FML18] *...list the main side effects of medicines, i.e. effects that are not intended but that could occur (headaches, nausea, diarrhea, dizziness, etc.).*

No items were removed from the Interactive or Critical domains, as all demonstrated acceptable fit. Infit mean square values for the retained items fell within or close to the acceptable range (0.7–1.3) across all subscales, with only minor deviations in the Functional domain even after item removal.

Plots of infit mean-square values for the Functional, Interactive, and Critical items are provided in the supplementary material (Appendix B). For the Critical subscale, visual inspection revealed two distinct clusters— CML36 to CML47 and CML48 to CML56 — which suggest the presence of a secondary dimension.

### 3.5 Item hierarchy and calibration

The item separation indices and reliability coefficients for the Functional (Item separation index= 7.20, reliability= 0.98), Interactive (Item separation index= 5.08, reliability= 0.96), and Critical (Item separation index= 13.14, reliability= 0.99) subscales exceeded recommended thresholds (Item separation index ≥ 2.0; reliability ≥ 0.70), indicating stable and consistent item hierarchies within each dimension of medication literacy. These results also confirm that the sample size was sufficient to ensure reliable calibration estimates.

However, the Wright maps (Appendix C) presented complementary information on item–person targeting. The Functional subscale revealed good alignment between item difficulty and participant ability, supporting satisfactory measurement precision. In contrast, the Interactive subscale contained items that were generally too easy for the sample, while the Critical subscale included items that were too difficult for most participants. Both patterns reflect suboptimal targeting, with items failing to cover the full range of abilities observed, thereby reducing measurement efficiency. These findings may also reflect a lack of heterogeneity of the study sample, which could have limited the variability in medication literacy skills. Future studies involving more heterogeneous populations could clarify whether these targeting patterns are sample-specific or instrument-related.

In summary, while the high item separation and reliability indices confirmed that the MED-fLAG has a stable and reproducible item hierarchy, the Wright maps underscored mismatches between item difficulty and participant ability—particularly in the Interactive and Critical domains.

### 3.6 Person-separation reliability (discrimination)

The person-separation indices and reliability coefficient findings for the Functional (Person-separation index= 1.80, reliability= 0.76), Interactive (Person-separation index= 1.53, reliability= 0.70), and Critical (Person-separation index= 1.87, reliability= 0.78) subscales met the minimum acceptable reliability threshold but indicated limited discrimination. In Rasch terms, Person-separation index values below 2.0 indicate that the MED-fLAG subscales could distinguish participants only to a limited extent, separating them into roughly one or two groups rather than across a full range of medication literacy levels. This restricted separation may reflect suboptimal item targeting and/or limited variability in the study sample. Refining item difficulty and including a more heterogeneous population in future validation work could enhance person separation and measurement precision.

### 3.7 Differential item functioning, by language

We conducted differential item functioning analyses to evaluate equivalence across language groups (French- vs. Dutch-speaking participants). In the Functional Medication Literacy subscale, one item (FML10) demonstrated moderate differential item functioning (DIF contrast= 0.82 logits), while another item (FML1) revealed statistically significant differential item functioning with a small contrast (0.30 logits), indicating negligible practical impact.

In the Interactive Medication Literacy subscale, two items (IML28 and IML30) demonstrated both statistically significant and practically meaningful differential item functioning (differential item functioning contrasts= 0.57 and 0.87 logits, respectively). Two additional items (IML25 and IML33) demonstrated statistically significant differential item functioning but with contrasts below the recommended threshold of 0.5 logits.

The Critical Medication Literacy subscale displayed the most pronounced cross-linguistic differences, with 14 items demonstrating statistically significant differential item functioning, of which six items (CML40, CML49, CML50, CML51, CML55, and CML56) exceeded the recommended threshold of 0.5 logits, indicating practically meaningful differential item functioning. The plots presented in Appendix D identify the specific items responsible for these cross-linguistic differences and point to priorities for future cross-language invariance testing.

### 3.8 Interpretability of the MED-fLAG by subscale

Regarding score interpretability, normalized z-scores on a 100-point scale showed that participants scored higher on the Functional (Mean= 77.13; Median= 84.2) and Interactive (Mean= 77.17; Median= 85.4) subscales, while scores were notably lower on the Critical subscale (Mean= 52.43; Median= 53.2). A ceiling effect was observed in 12% of participants for the Functional subscale, remaining below the conventional 15% threshold. Floor effects were negligible across all three dimensions (<1%), indicating that very few participants scored at the lowest levels.

The score distributions also suggest that the study sample was relatively homogeneous, with most participants demonstrating rather good functional and interactive medication literacy. This limited variability may have contributed to the small ceiling effect observed and could partly explain the moderate person separation indices reported above.

## 4 Discussion

This study aimed to examine the psychometric properties of the MED-fLAG, a multidimensional scale recently developed to assess medication literacy in older adults. Overall, the findings based on Rasch analyses offer preliminary evidence of satisfactory measurement properties of the MED-fLAG while also emphasizing several areas for improvement. Specifically, the analyses identified several issues in the Critical subscale, in person-separation indices, and in cross-language equivalence, indicating priorities for refinement in future versions of the MED-fLAG.

**Unidimensionality**

Findings generally support the structural validity of the MED-fLAG, with the Functional and Interactive subscales demonstrating clear unidimensionality. In contrast, the Critical subscale demonstrated signs of multidimensionality, likely due to the use of mixed response formats: whereas most items in the scale assessed perceived difficulty, several Critical items relied on frequency-based response options. Previous research has revealed that heterogeneous formats can elicit different cognitive processes and reduce construct coherence (Blair and Burton, 1987, Schwarz et al., 2012). This inconsistency may have introduced measurement noise and weakened the psychometric performance of the Critical subscale.

The Critical items based on frequency-based response options are more likely to assess practical actions rather than cognitive understanding or judgment (e.g., [CML42] “…*use a treatment plan that describes the medicines that need to be taken*”; [CML44] “…*use a pillbox that you prepare yourself for several days, without the help of a health professional*”. From a conceptual standpoint, this aligns more closely with the concept of medication self-management than with medication literacy, which focuses primarily on cognitive and social skills (Gentizon et al., 2022a). While medication self-management and medication literacy may share conceptual overlap (Mortelmans et al., 2024), self-management more explicitly integrates emotional aspects as well as physical abilities and behaviours related to medication use (Bailey et al., 2013, Cadel et al., 2021). In this context, frequency-based CML items (CML36–CML48) may align more closely with medication self-management. They may therefore deviate from the narrower definition of medication literacy, which focuses on cognitive and social skills. This supports the need to conceptually revise the Critical subscale, ensuring that it reflects the intended construct and uses consistent response formats. Such refinement could strengthen unidimensionality, improve measurement precision, and enhance the interpretability of Critical Medication Literacy among older adults.

Taken together, these results align with the existing theoretical frameworks distinguishing three domains of medication literacy – Functional, Interactive, and Critical (Emmerton et al., 2012, Pantuzza et al., 2020, Vervloet et al., 2018, Gentizon et al., 2022a). While most existing instruments focus primarily on functional skills and place little emphasis on interactive or critical aspects (Gentizon et al., 2021), our results emphasize both the central role of functional skills and the added value of incorporating interactive and critical dimensions to achieve a more comprehensive understanding of medication literacy.

**Discrimination**

The person-separation indices indicated limited discrimination, indicating that the MED-fLAG could not clearly distinguish participants across the full range of medication literacy levels. This limitation likely results from both item targeting and sample characteristics. Although the study successfully recruited a socio-demographically diverse group of older adults—with variability in age, education, and living situation—variability in medication literacy levels was relatively limited. Subscale scores were generally high, particularly in the Functional and Interactive dimensions, which may have contributed to the moderate Person-separation indices observed.

A notable finding was that, despite high Functional and Interactive scores, 64.8% of Dutch-speaking participants reported difficulties with medication self-management. We cannot exclude the possibility that self-reporting and data collection shortly before hospital discharge may have introduced motivational or social desirability biases when responding to the MED-fLAG, potentially leading participants to underreport their difficulties. Such biases are particularly relevant for individuals with low literacy, who may experience stigma and adopt coping mechanisms to conceal their difficulties (Baker et al., 1996, Kripalani et al., 2006). Future studies should include participants with a wider range of medication literacy levels. In particular, individuals with documented medication-related problems or adverse events could increase variance and improve person-separation indices (Bond and Fox, 2013).

Refining the MED-fLAG items to better cover the full spectrum of medication literacy would improve the sensitivity of the subscales to mild, moderate, and severe limitations. As suggested by others (Osborne et al., 2013), this revision could draw on the revised Bloom’s taxonomy. This framework organises cognitive tasks along two dimensions of increasing complexity. The first dimension describes levels of knowledge—factual, conceptual, procedural, and meta-cognitive—while the second outlines progressively more demanding cognitive processes, including remembering, understanding, applying, analysing, evaluating, and creating (Anderson and Krathwohl, 2001, Krathwohl, 2002). For example, in the Interactive subscale, more challenging items could include: “…*apply instructions that healthcare professionals gave you about your medications*” [Applying, executing] or “…*express your expectations and concerns about the medicines you are managing (e.g., schedules, number of medicines*)” [Making judgements; critiquing]. Conversely, easier items in the Critical subscale could be: “…*list sources of information that can be considered reliable*” [Conceptual knowledge], “…*know what to do when encountering a problem with medications (e.g., forgetting, experiencing unintended effects)*” [Procedural knowledge], or “…*identify situations where you need to seek additional information*” [Self-knowledge].

**Response format and scale optimization**

Another potential explanation for the limited discrimination relates to the number of response options. Researchers have long debated how the number and clarity of response options affect psychometric performance (Abulela and Khalaf, 2024, Lozano et al., 2008, Preston and Colman, 2000). Scales with too few or poorly differentiated response categories often yield lower reliability and disordered thresholds, thereby reducing their ability to discriminate between respondents (Lozano et al., 2008, Elsworth et al., 2016, Osborne et al., 2013). The current 4-point MED-fLAG response scale may include intermediate categories (“somewhat difficult” and “difficult”) that are insufficiently distinct.

One avenue for improvement would be to adopt a response format already validated in other literacy measures, such as the Health Literacy Questionnaire. The Health Literacy Questionnaire, which uses a 5-point scale (1= cannot do, 2= very difficult, 3= quite difficult, 4= quite easy, 5= very easy), has demonstrated robust psychometric performance in Rasch analyses across diverse populations (Slatyer et al., 2022, Bessing et al., 2022, Osborne et al., 2013). Adopting a similar format could improve the interpretability of MED-fLAG results. It could also facilitate comparisons with related constructs such as health literacy and strengthen its relevance for cross-population research. Future studies with the MED-fLAG should also include item threshold ordering analyses to verify the optimal functioning of response categories, as this was not performed in the present study.

**Cross-language validity**

Differential item functioning analyses revealed cross-linguistic differences across several items, particularly within the Critical subscale. While the Functional and Interactive dimensions revealed only a limited number of items with practically meaningful differential item functioning, the Critical subscale displayed more pronounced cross-language differences, with multiple items exceeding the recommended threshold for meaningful differential item functioning. Sociodemographic differences between language groups (Dutch versus French)—such as age, living situation, and education—may have influenced item interpretation and performance.

An additional explanation for the cross-language differences observed may lie in the translation process itself. Although a forward–backward translation procedure was performed, the Dutch version of the MED-fLAG was not subjected to formal cognitive debriefing with target users. International guidelines for the translation of patient-reported outcome measures recommend cognitive interviewing to verify that translated items are interpreted as intended and that conceptual equivalence with the original version is maintained (Prinsen et al., 2018). Without this step, subtle linguistic or cultural differences in how items are understood may remain undetected and can contribute to differential item functioning. The substantial differential item functioning observed in several Critical Medication Literacy items may therefore partly reflect differences in interpretation rather than true differences in medication literacy levels between language groups.

### 4.1 Strengths and limitations

One of the main strengths of this study is that it is the first to report psychometric results for a medication-related measure specifically designed for older populations using modern Rasch analyses, as recommended by several authors (Nguyen et al., 2015, Sauceda et al., 2012). By applying Rasch modelling, this study was able to identify psychometric properties of the MED-fLAG that extend beyond the scope of classical test theory, including item calibration, item–person targeting, and cross-language item functioning. These results present a nuanced, item-level understanding of the scale’s performance and offer clear directions for refining the MED-fLAG to enhance its precision and clinical applicability in ageing populations.

Despite these strengths, several limitations should be acknowledged. Psychometric guidelines recommend that cross-cultural validation be conducted only after sufficient evidence has been established for the original language version (Mokkink et al., 2016, Prinsen et al., 2018). In the present study, data from French- and Dutch-speaking participants were pooled to improve the stability of item calibration given the available sample size. Although this approach increased statistical power, it represents a deviation from recommended validation procedures and requires cautious interpretation of the findings. The substantial differential item functioning observed in the Critical subscale further underlines the need to interpret pooled calibration results carefully.

In addition, although the Dutch version of the MED-fLAG was translated using a forward–backward procedure, formal cognitive debriefing with target users was not conducted. Cognitive interviewing is recommended in Patient-Reported Outcome Measure translation guidelines (Prinsen et al., 2018) to ensure conceptual and linguistic equivalence across language versions, and its absence may have contributed to some of the cross-language differences observed. Future studies should therefore aim to formally assess cross-language measurement invariance using recommended approaches such as anchored Rasch analyses or separate calibrations followed by linking procedures and incorporate structured cognitive interviews during translation and adaptation processes.

### 4.2 Implications for further research with the MED-fLAG

Overall, these psychometric findings inform several directions for the further development and validation of the MED-fLAG. While the Functional and Interactive domains demonstrated satisfactory measurement properties, the Critical domain—which is conceptually more innovative—requires further conceptual and methodological clarification to enhance its validity and reliability. Particular attention should be paid to the Critical Medication Literacy subscale, where cross-language differences were more pronounced, indicating that some items may require additional conceptual clarification or cross-cultural adaptation.

In addition, practical aspects of the instrument also warrant attention. With 56 items, the current MED-fLAG may be too time-consuming for routine clinical use. In a previous study that we conducted (Gentizon et al., 2022b), the MED-fLAG demonstrated good acceptability, although its length was identified as a potential challenge for routine clinical use. Future research should therefore explore the development of a shorter version of the MED-fLAG, or a screening form that could rapidly identify individuals at risk of medication literacy difficulties. Depending on the primary purpose of the MED-fLAG in clinical practice, a brief screening version could be used in routine care to identify patients who may require additional support, while the full MED-fLAG could be retained for more comprehensive assessment. In this regard, Rasch analyses offer a robust framework for such optimization by identifying the most informative items across the continuum of medication literacy. Barriers to implementing Patient-Reported Outcome Measures in clinical practice persist (Amini et al., 2021). Achieving an appropriate balance between comprehensive assessment and practical feasibility will therefore be essential for the successful implementation of the MED-fLAG. In this regard, the use of implementation science frameworks could further support its integration into clinical practice (Stover et al., 2021).

Future psychometric studies should also focus on strengthening the construct validity of the MED-fLAG, including assessments of convergent and discriminant validity (Jang et al., 2019, Weinert et al., 2019, Koster et al., 2018), responsiveness to change, and associations with clinically relevant outcomes such as medication adherence or hospital readmission (Prinsen et al., 2018). Establishing these properties would further support the clinical and research utility of the instrument.

Finally, future studies should aim to increase the diversity of the samples recruited. Including larger and more heterogeneous populations—such as individuals with low literacy, documented medication-related problems, or adverse drug events—could improve the scale’s measurement properties and increase variability in medication literacy levels. Testing the instrument in different settings, such as primary care and home care, would also help assess its ecological validity. Furthermore, as the MED-fLAG was initially designed to assess medication literacy in both older adults and informal caregivers, future research should evaluate its psychometric performance in caregiver populations. This step is particularly relevant given that, to date, no health literacy self-report scale for older adults has been validated in caregivers (Slatyer et al., 2022).

## 5 Conclusions

The MED-fLAG is a newly developed, geriatric-specific, multidimensional scale for assessing medication literacy, with promising initial evidence of validity and reliability. This study provides the first Rasch-based psychometric evaluation of the MED-fLAG, supporting its multidimensional conceptual foundation while also identifying several areas for refinement, particularly within the Critical subscale and in cross-language equivalence.

In the future, the MED-fLAG could support nurses and other healthcare professionals in formally assessing medication literacy among older adults and informal caregivers, ensuring that prescriptions and education are tailored to individual abilities—especially during care transitions, when medication-related problems are most likely to occur (Alqenae et al., 2020, Daliri et al., 2019, Tomlinson et al., 2020). By providing structured insight into patients’ and caregivers’ medication literacy levels, the MED-fLAG could equip nurses with essential information to deliver individualised interventions (e.g., educational strategies, simplification of prescriptions) and help prevent medication-related problems. However, further psychometric testing of a revised version—particularly in diverse populations and care settings—is required before the MED-fLAG can be confidently implemented in clinical practice.

## Ethics approval and consent to participate

This project was approved by the Ethics Committee of the Canton of Vaud, Switzerland (CERVD 202301228) and the ethics committees of the participating hospitals in Belgium (ECOG099 and B3002022000169) and the Netherlands (ID2021075 and METC20212023).

## Declaration of generative AI and AI-assisted technologies in the manuscript preparation process

The authors used ChatGPT to assist in improving the English language and clarity of expression. After using this tool, the authors carefully reviewed and edited the content as necessary. The authors take full responsibility for the final content of the manuscript.

## Funding statement

This study was conducted as part of a Master’s thesis in Nursing Sciences at the University of Lausanne and the University of Antwerp. This research did not receive any specific grant from funding agencies in the public, commercial, or not-for-profit sectors.

## CRediT authorship contribution statement

**Jenny Gentizon:** Writing – original draft, Validation, Supervision, Methodology, Conceptualization. **Laura Mortelmans:** Writing – review & editing, Supervision, Investigation, Conceptualization. **Nadège Tapsoba:** Writing – review & editing, Investigation, Conceptualization. **Nathalie Rebetez:** Writing – review & editing, Investigation. **Anne-Lore Bynens:** Writing – review & editing, Investigation, Conceptualization. **Carole Schol:** Writing – review & editing, Investigation, Conceptualization. **Louise Vanheusden:** Writing – review & editing, Investigation. **Anneleen Van De Weyer:** Writing – review & editing, Investigation, Conceptualization. **Roger Hilfiker:** Writing – review & editing, Software, Methodology, Formal analysis, Data curation. **Tinne Dilles:** Writing – review & editing, Supervision, Resources, Conceptualization. **Cedric Mabire:** Writing – review & editing, Supervision, Resources, Conceptualization.

## Declaration of competing interest

The authors declare that they have no known competing financial interests or personal relationships that could have appeared to influence the work reported in this paper.

## Data Availability

The data that support the findings of this study are available from the corresponding author upon reasonable request.

## Appendix Supplementary materials

Image, application 1
