# Supplementary material for: Psychometric evaluation of the MEDication Literacy Assessment in Geriatric Patients and Informal Caregivers (MED-fLAG) instrument using Rasch analyses in a sample of hospitalised older adults
Source: Int J Nurs Stud Adv. 2026 Jun 24;11:100610. doi: 10.1016/j.ijnsa.2026.100610 (PMC13325989; doi:10.1016/j.ijnsa.2026.100610)
Supplement: Supplementary file 1 [file mmc1.docx]

**Appendix A**

**The 56-item MED-fLAG (Medication Literacy Assessment in Geriatric patients and informal Caregivers) – version used in the psychometric validation study**

(Free translation from French)

For use, translation, or adaptation, please contact the corresponding author.

| **Functional medication literacy** [FML] is the degree to which patients or their informal caregivers: a) have a basic knowledge about the medications; b) know the purpose; c) understand the instructions related to the preparation and administration; d) are able prepare correct dosages (i.e., calculating); and e) are able to monitor of the effects and observe precautions. | | |
| --- | --- | --- |
|  |  |  |
| **Suppose you have to describe what you know about the medication you manage, and what you have understood about the instructions.  How difficult is it for you to:**  (4 = Not difficult at all; 3 = Somewhat difficult; 2 = Difficult; 1 = Very difficult or impossible; NA = Not applicable to my situation) | | |
| **Knowing medication basic information (6)** | **FML1** | ...list the names of all the medicines you manage, both prescription and non-prescription (either by heart or with the help of a support such as a prescription list) |
|  | **FML2** | ...list the names of the natural remedies you manage, e.g. herbal treatments, homeopathy, dietary supplements (by heart or with a support such as a prescription list) |
|  | **FML3** | ...describe medicines by their appearance (colour and shape) |
|  | **FML4** | ...tell which medicines are pills, ointments, drops, or are used for injections or inhalations |
|  | **FML5** | ...tell how medicines should be stored, where to keep them (including after opening for creams, drops etc.) |
|  | **FML6** | ...give the names of the various physicians or other health professionals who have prescribed the medicines you manage |
| **Knowing the purpose of the medication treatment (4)** | **FML7** | ...explain in your own words why each medicine has been prescribed |
|  | **FML8** | ...explain in your own words what effect each medicine is expected to have |
|  | **FML9** | ...describe the problems that might occur if the medicines are not taken, or if they are taken differently from the instructions that have been given |
|  | **FML10** | ...say whether the list of current medicines includes any PRN medicines (i.e. to be taken only when needed) |
| **Understanding preparation and administration instructions (5)** | **FML11** | ...tell which medicine should be taken before, during or after a meal |
|  | **FML12** | ...tell the schedule (hours) for taking each medicine |
|  | **FML13** | ...say how many times a day each medicine should be taken |
|  | **FML14** | …say how long each medicine should be taken |
|  | **FML15** | …tell the dosage of each medicine (by heart or with the help of a support such as a prescription list) |
| **Preparing correct dosages by calculating (numeracy) (2)** | **FML16** | ...prepare the correct dosage of medicines, e.g. counting the number of tablets, the number of drops, or using a graduated syringe (or measuring cup) |
|  | **FML17** | ...count how many medicines need to be prepared to cover several days of treatment (e.g. when going on holiday or away from home) |
| **Monitoring effects and observing precautions (5)** | **FML18** | ...list the main side effects of medicines, i.e. effects that are not intended but that could occur (headaches, nausea, diarrhea, dizziness, etc.) |
|  | **FML19** | ...tell whether any of the medicines you manage may have effects that require monitoring from your part |
|  | **FML20** | ...tell whether certain medicines are incompatible with those you are managing (including natural remedies and herbal treatments) |
|  | **FML21** | ...tell whether certain foods or drinks should be avoided or are prohibited with the medicines you manage (alcohol, grapefruit juice, lactose-based foods, etc.) |
|  | **FML22** | ...tell whether any of the medicines you manage require special precautions (e.g. avoid driving a car, exposure to the sun, hygiene precautions) |
| **Interactive medication literacy** [IML] is the degree to which individuals: a) ask for clarification and understand explanations given by healthcare professionals; b) provide medication-related information; and c) inform about medication-related difficulties and problems. | | |
| **Suppose you have to describe your ease of communication with health professionals about the medicines you manage How difficult is it for you to:**  (4 = Not difficult at all; 3 = Somewhat difficult; 2 = Difficult; 1 = Very difficult or impossible; NA = Not applicable to my situation) | | |
| **Asking for clarification, advice and understanding explanations (3)** | **IML23** | ...ask health professionals for additional information about the medicines you manage (precautions to take, risks and benefits, changes to the current medicine list, etc.) |
|  | **IML24** | ...ask health professionals for advice about natural remedies, e.g. herbal remedies, homeopathy and food supplements |
|  | **IML25** | ...understand information about medicines given by health professionals |
| **Providing medication-related information, including preferences (5)** | **IML26** | ...describe the latest changes that have been made in the list of current medicines (new medicines initiated, those removed, changes in dosage) |
|  | **IML27** | ...express your expectations, give your opinion about the medicines you are managing (e.g. about the schedules (hours), the number of medicines, etc.) |
|  | **IML28** | ...express your interest in natural remedies such as herbal remedies, homeopathy, food supplements |
|  | **IML29** | ... provide information about the effects observed, what has been experienced that you think could be associated with the medicines |
|  | **IML30** | ...give information about known allergies or poorly tolerated drugs (including contrast media, iodine, etc.) |
| **Informing about medication-related difficulties, and problems (5)** | **IML31** | ... discuss any difficulties in following the medication plan, or in following the instructions |
|  | **IML32** | ... report about mistakes, omissions or other problems with medication that have happened in the last few weeks (e.g. wrong dosage, wrong time of day, getting two medicines mixed up) |
|  | **IML33** | ...tell a health professional if you have stopped or changed the dosage of a medicine, or if you are thinking of doing so |
|  | **IML34** | ...talk about conflicting information you have received about the medicines you are managing (information received from another health professional or someone you know) |
|  | **IML35** | ...seek professional advice about information about medicines that you have found in the media, advertisements, health magazines or social networks |
| **Critical medication literacy** [CML] is the degree to which individuals: a) seek reliable medication-related information; b) set up strategies and practical means to integrate medication taking in a daily routine; and c) have the control over their medication and adjust when the situation changes. | | |
| **About your personal habits and the ways in which you find information about medicines. Do you usually:** (4 = Always; 3 = Often; 2 = Sometimes; 1 = Never; NA = Not applicable to my situation) | | |
| **Seeking reliable sources of information and being critical about the information found (5)** | **CML36** | ...read the package insert given with the medicine box |
|  | **CML37** | ...read patient leaflets about medicines (found in pharmacies, waiting rooms or given by a health professional) |
|  | **CML38** | ...seek advice from a health professional before taking any over-the-counter medicines, including natural herbal remedies, homeopathy and food supplements |
|  | **CML39** | ...seek information when a new medicine has been prescribed |
|  | **CML40** | ...question the reliability of information about medicines that you find in the media, advertisements, health magazines or social networks |
| **Regarding your routines and the strategies you use to support you in managing medicines Do you usually:** (4 = Always; 3 = Often; 2 = Sometimes; 1 = Never; NA = Not applicable to my situation) | | |
| **Setting up strategies and routines to facilitate the integration of medicines in a daily routine (8)** | **CML41** | …carry a record of the medicines you manage (in your wallet, on your phone) |
|  | **CML42** | ...use a treatment plan that describes the medicines that need to be taken |
|  | **CML43** | ...use a routine, some strategies that allow you to check that the medicines have not been forgotten (alert on the phone, note each time the medicines are taken, ask a family member to check, etc.) |
|  | **CML44** | ...use a pillbox that you prepare yourself for several days, without the help of a health professional |
|  | **CML45** | ...take some medicines with you when you leave home (in case of unexpected events) |
|  | **CML46** | ...write down questions about the medicines you want to ask at the next visit to the physician or other health professional (in a notebook, calendar, etc.) |
|  | **CML47** | ...keep a record of the decisions that have been made concerning the medicines you manage (new medicines initiated, those removed, changes in dosage) |
|  | **CML48** | ...go to the same pharmacy to get medicines (having the medication file in a same and unique pharmacy) |
| **Suppose you have to describe how taking medication fits into your life organisation How difficult is it for you to:** (4 = Not difficult at all; 3 = Somewhat difficult; 2 = Difficult; 1 = Very difficult or impossible; NA = Not applicable to my situation) | | |
| **Having control over the management of medication including when the situation changes (prescription changes, occurrence of problems) (8)** | **CML49** | ...set up daily routines so that you don't forget to take your medication (e.g. medication at the bedside, in the bathroom, on the kitchen table) |
|  | **CML50** | ...know when to schedule a prescription renewal by the physician or other health professionals |
|  | **CML51** | ...organise the stock of medicines and know when to go to the pharmacy to get a refill or to get new medicines after prescription change |
|  | **CML52** | ...adapt your daily routines after the list of medicines has changed (e.g. after hospitalisation) |
|  | **CML53** | …get answers to questions you have about medicines, obtain advice |
|  | **CML54** | ...get help from your family or people around you if you have difficulties with medicines |
|  | **CML55** | ...get help from health professionals if you have problems with medicines |
|  | **CML56** | ......know which healthcare professional to contact if you have problems with the medicines you manage |

**Appendix B**

**Item** **fit statistics for the Functional [FML], Interactive [IML] and [CML] items, based on infit and mean-square values**

The table below presents Rasch item fit statistics for the Functional Medication Literacy (FML) subscale based on the initial calibration including all 22 items. Acceptable item fit was defined as infit mean-square statistics between 0.7 and 1.3 (Bond & Fox, 2013; Linacre & Wright, 2000; Smith, 1995). Items marked with an asterisk (*) indicate those exceeding the recommended infit threshold and subsequently removed from the final model.

**Table B1**

**Item fit statistics for the Functional Medication Literacy (FML) subscale, with 22 items**

| **Item** | **Measure (logits)** | **SE** | **Infit MNSQ** | **Outfit MNSQ** | **PTMEA Corr.** | **Interpretation** |
| --- | --- | --- | --- | --- | --- | --- |
| FML1 | 0.56 | 0.06 | 1.06 | 1.11 | 0.65 | Acceptable fit |
| **FML2*** | -0.03 | 0.06 | 0.65 | 0.62 | 0.71 | Overfit – overly predictable responses (item removed) |
| FML3 | 0.03 | 0.06 | 1.05 | 1.08 | 0.60 | Acceptable fit |
| FML4 | -0.59 | 0.07 | 1.00 | 0.93 | 0.54 | Acceptable fit |
| FML5 | -0.62 | 0.07 | 0.96 | 0.98 | 0.54 | Acceptable fit |
| FML6 | 0.04 | 0.06 | 1.23 | 1.48 | 0.55 | Slight outfit misfit (item retained) |
| FML7 | -0.06 | 0.06 | 0.91 | 0.88 | 0.63 | Acceptable fit |
| FML8 | 0.17 | 0.06 | 0.98 | 0.85 | 0.64 | Acceptable fit |
| FML9 | 0.54 | 0.06 | 1.20 | 1.30 | 0.62 | Acceptable fit |
| FML10 | -0.06 | 0.06 | 0.88 | 0.68 | 0.61 | Acceptable fit |
| FML11 | -0.54 | 0.07 | 0.97 | 1.20 | 0.53 | Acceptable fit |
| FML12 | -0.71 | 0.07 | 0.77 | 0.65 | 0.56 | Acceptable fit |
| **FML13*** | -0.65 | 0.07 | 0.62 | 0.37 | 0.56 | Overfit – potential redundancy (item removed) |
| FML14 | -0.34 | 0.07 | 0.84 | 0.86 | 0.59 | Acceptable fit |
| FML15 | 0.67 | 0.05 | 1.29 | 1.39 | 0.62 | Slight outfit misfit (item retained) |
| FML16 | -0.46 | 0.07 | 0.77 | 0.83 | 0.59 | Acceptable fit |
| FML17 | -0.48 | 0.07 | 0.72 | 0.53 | 0.60 | Acceptable fit |
| **FML18*** | 1.36 | 0.06 | 1.66 | 1.82 | 0.60 | Underfit – inconsistent responses (item removed) |
| FML19 | 0.33 | 0.06 | 0.96 | 0.88 | 0.65 | Acceptable fit |
| FML20 | 0.91 | 0.05 | 1.29 | 1.34 | 0.64 | Slight outfit misfit (item retained) |
| FML21 | 0.07 | 0.06 | 1.25 | 1.36 | 0.56 | Slight outfit misfit (item retained) |
| FML22 | -0.15 | 0.06 | 0.83 | 0.68 | 0.62 | Acceptable fit |

**
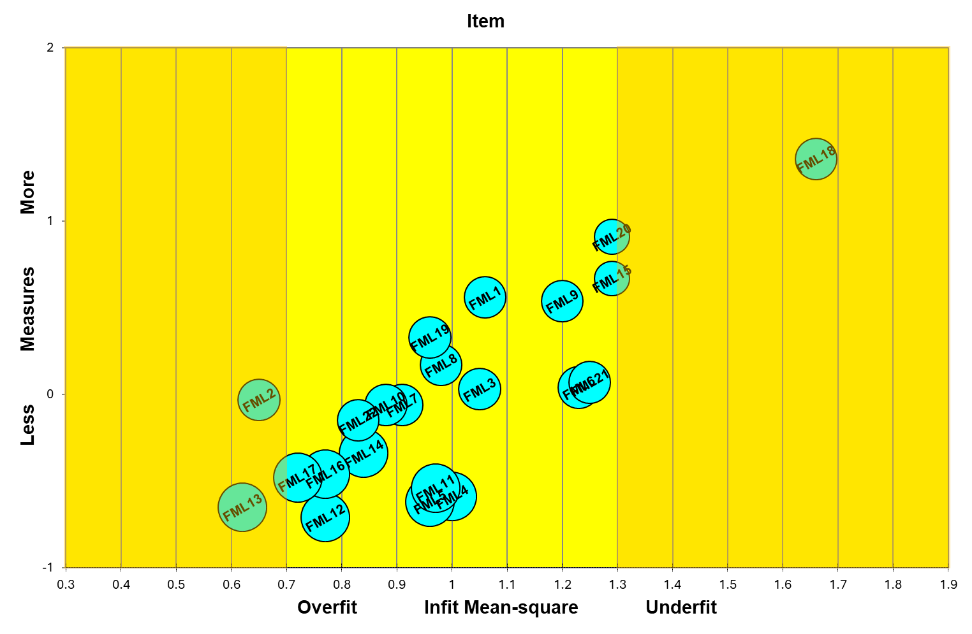
**

*Figure 1.* Plot of infit mean-square statistics for the Functional Medication Literacy items before item removal.

The following table presents the Rasch item fit statistics for the Functional Medication Literacy (FML) subscale after removal of the three items showing problematic infit statistics (FML2, FML13, and FML18). The model was recalibrated using the remaining 19 items to evaluate item fit within the refined Functional subscale.

**Table B2**

**Item fit statistics for the Functional Medication Literacy (FML) subscale, with 19 items**

| **Item** | **Measure (logits)** | **SE** | **Infit MNSQ** | **Outfit MNSQ** | **PTMEA Corr.** | **Interpretation** |
| --- | --- | --- | --- | --- | --- | --- |
| FML15 | 0.71 | 0.05 | 1.34 | 1.47 | 0.62 | Slight misfit – retained |
| FML6 | 0.07 | 0.06 | 1.21 | 1.41 | 0.57 | Slight outfit misfit – retained |
| FML20 | 0.95 | 0.06 | 1.32 | 1.37 | 0.65 | Slight misfit – retained |
| FML9 | 0.58 | 0.06 | 1.22 | 1.33 | 0.63 | Acceptable fit |
| FML21 | 0.10 | 0.06 | 1.21 | 1.28 | 0.58 | Acceptable fit |
| FML1 | 0.60 | 0.06 | 1.11 | 1.21 | 0.65 | Acceptable fit |
| FML11 | -0.50 | 0.07 | 0.96 | 1.14 | 0.54 | Acceptable fit |
| FML3 | 0.07 | 0.06 | 1.06 | 1.12 | 0.60 | Acceptable fit |
| FML4 | -0.56 | 0.07 | 1.00 | 0.88 | 0.55 | Acceptable fit |
| FML19 | 0.37 | 0.06 | 0.99 | 0.98 | 0.65 | Acceptable fit |
| FML8 | 0.20 | 0.06 | 0.96 | 0.85 | 0.66 | Acceptable fit |
| FML5 | -0.58 | 0.07 | 0.93 | 0.90 | 0.55 | Acceptable fit |
| FML7 | -0.03 | 0.06 | 0.90 | 0.86 | 0.64 | Acceptable fit |
| FML10 | -0.03 | 0.06 | 0.86 | 0.65 | 0.62 | Acceptable fit |
| FML14 | -0.30 | 0.07 | 0.82 | 0.82 | 0.60 | Acceptable fit |
| FML22 | -0.11 | 0.06 | 0.81 | 0.67 | 0.64 | Acceptable fit |
| FML16 | -0.43 | 0.07 | 0.78 | 0.76 | 0.60 | Acceptable fit |
| FML12 | -0.67 | 0.07 | 0.77 | 0.65 | 0.57 | Acceptable fit |
| FML17 | -0.44 | 0.07 | 0.73 | 0.54 | 0.60 | Acceptable fit |

The table below presents Rasch item fit statistics for the Interactive Medication Literacy (IML) subscale based on the calibration including all 13 items. Infit and outfit mean-square values are reported for each item to evaluate their fit to the Partial Credit Model.

**Table B3**

**Item fit statistics for the Interactive Medication Literacy (IML) subscale, including 13 items**

| **Item** | **Measure (logits)** | **SE** | **Infit MNSQ** | **Outfit MNSQ** | **PTMEA Corr.** | **Interpretation** |
| --- | --- | --- | --- | --- | --- | --- |
| IML26 | 0.59 | 0.07 | 1.17 | 1.27 | 0.71 | Acceptable fit |
| IML28 | 0.68 | 0.07 | 1.25 | 1.24 | 0.73 | Acceptable fit |
| IML29 | 0.47 | 0.07 | 1.15 | 1.24 | 0.72 | Acceptable fit |
| IML30 | -0.21 | 0.08 | 1.06 | 1.17 | 0.66 | Acceptable fit |
| IML23 | -0.60 | 0.08 | 1.03 | 1.15 | 0.65 | Acceptable fit |
| IML25 | -0.15 | 0.08 | 0.97 | 1.12 | 0.69 | Acceptable fit |
| IML32 | -0.27 | 0.08 | 0.94 | 1.12 | 0.67 | Acceptable fit |
| IML34 | 0.23 | 0.07 | 0.94 | 1.10 | 0.71 | Acceptable fit |
| IML24 | 0.14 | 0.08 | 0.99 | 0.94 | 0.74 | Acceptable fit |
| IML35 | 0.22 | 0.07 | 0.93 | 0.83 | 0.73 | Acceptable fit |
| IML27 | -0.17 | 0.08 | 0.88 | 0.78 | 0.69 | Acceptable fit |
| IML31 | -0.58 | 0.08 | 0.85 | 0.77 | 0.68 | Acceptable fit |
| IML33 | -0.35 | 0.08 | 0.80 | 0.77 | 0.70 | Acceptable fit |

**
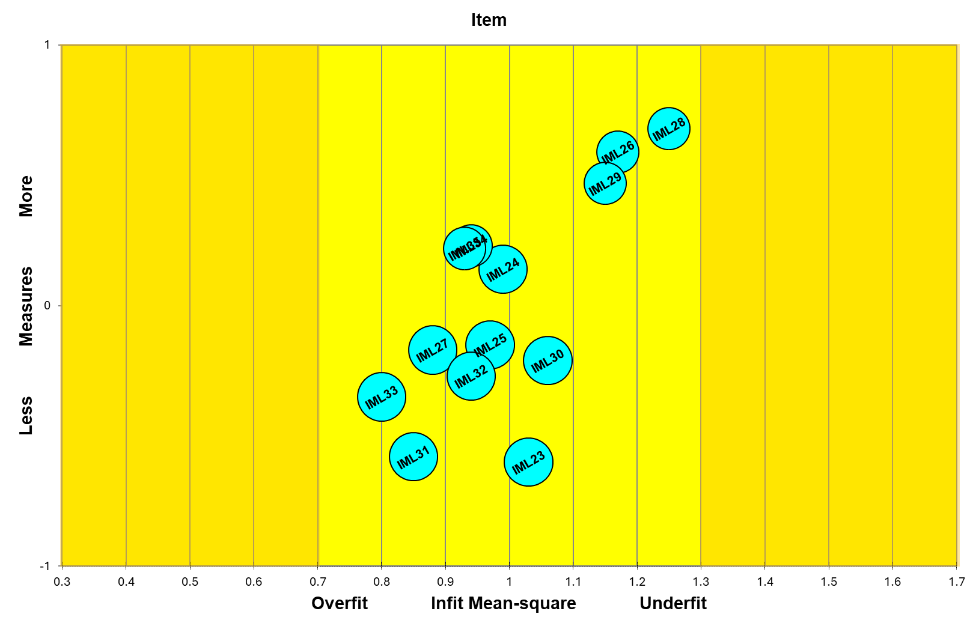
**

*Figure 2.* Plot of infit mean-square statistics for the Interactive Medication Literacy items

The table below presents Rasch item fit statistics (infit and outfit mean-square values) for the 21 items of the Critical Medication Literacy (CML) subscale.

**Table B4**

**Item fit statistics for the Critical Medication Literacy (FML) subscale, including 21 items**

| **Item** | **Measure (logits)** | **SE** | **Infit MNSQ** | **Outfit MNSQ** | **PTMEA Corr.** | **Interpretation** |
| --- | --- | --- | --- | --- | --- | --- |
| CML43 | 0.69 | 0.04 | 1.16 | 1.43 | 0.43 | Acceptable fit |
| CML45 | 0.39 | 0.04 | 1.21 | 1.37 | 0.40 | Acceptable fit |
| CML44 | 0.64 | 0.04 | 1.20 | 1.36 | 0.43 | Acceptable fit |
| CML36 | 0.43 | 0.04 | 1.17 | 1.27 | 0.40 | Acceptable fit |
| CML41 | 0.59 | 0.04 | 1.09 | 1.21 | 0.46 | Acceptable fit |
| CML39 | 0.81 | 0.04 | 1.11 | 1.13 | 0.46 | Acceptable fit |
| CML40 | 0.20 | 0.04 | 1.10 | 1.11 | 0.43 | Acceptable fit |
| CML46 | 0.59 | 0.04 | 1.06 | 1.09 | 0.47 | Acceptable fit |
| CML37 | 0.80 | 0.04 | 1.04 | 1.03 | 0.48 | Acceptable fit |
| CML54 | -0.54 | 0.06 | 0.85 | 1.04 | 0.45 | Acceptable fit |
| CML38 | 0.34 | 0.04 | 1.00 | 1.02 | 0.48 | Acceptable fit |
| CML42 | 0.66 | 0.04 | 0.93 | 0.93 | 0.53 | Acceptable fit |
| CML47 | 0.75 | 0.04 | 0.91 | 0.93 | 0.54 | Acceptable fit |
| CML48 | -0.87 | 0.06 | 0.92 | 0.87 | 0.41 | Acceptable fit |
| CML56 | -0.69 | 0.06 | 0.85 | 0.80 | 0.46 | Acceptable fit |
| CML55 | -0.60 | 0.06 | 0.82 | 0.79 | 0.48 | Acceptable fit |
| CML49 | -0.84 | 0.06 | 0.81 | 0.81 | 0.46 | Acceptable fit |
| CML53 | -0.88 | 0.06 | 0.77 | 0.78 | 0.49 | Acceptable fit |
| CML52 | -0.85 | 0.06 | 0.76 | 0.74 | 0.51 | Acceptable fit |
| CML51 | -0.93 | 0.07 | 0.73 | 0.63 | 0.50 | Acceptable fit |
| CML50 | -0.68 | 0.06 | 0.72 | 0.71 | 0.51 | Acceptable fit |

**
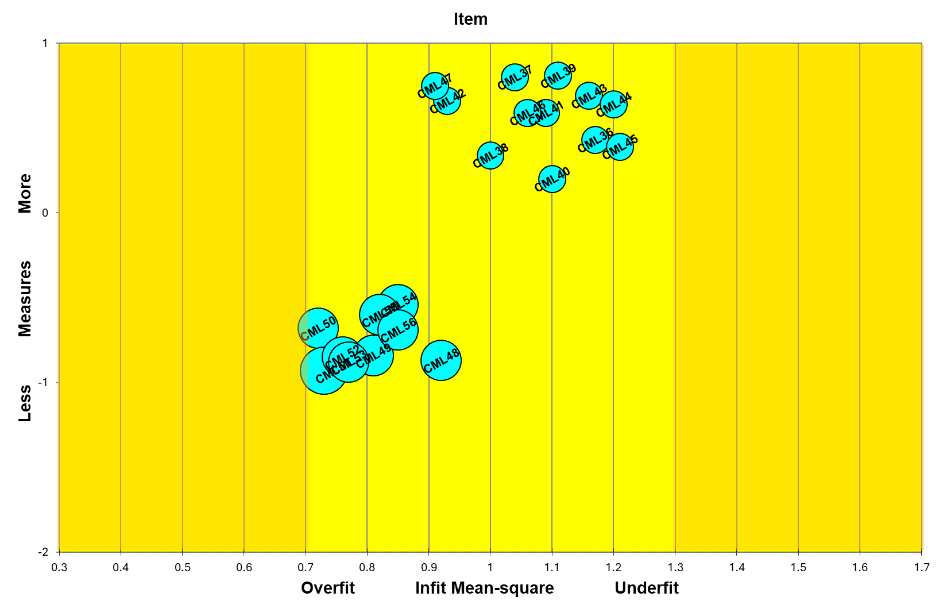
**

*Figure 3.* Plot of infit mean-square statistics for the Critical Medication Literacy items

**Appendix C**

**Wright maps illustrating person ability in blue, and item difficulty in red for (from left to right): the Functional, Interactive, and Critical Medication Literacy subscales of the MED-fLAG**

The Wright maps illustrate the alignment between item difficulty (red bars) and person ability (blue bars) for the Functional, Interactive, and Critical Medication Literacy subscales of the MED-fLAG.

The visual inspection of the Wright map for the Functional Medication Literacy subscale shows that most participants’ abilities are centred between +0.5 and +2.5 logits, indicating moderate-to-high functional medication literacy, which is congruent with the mean score (77.13, SD = 22.20). The item difficulties are generally well matched to the participants' ability levels. Items are clustered fairly closely together in difficulty, around the 0 to +1 logit range. This supports rather good measurement precision in the Functional subscale within the sample.


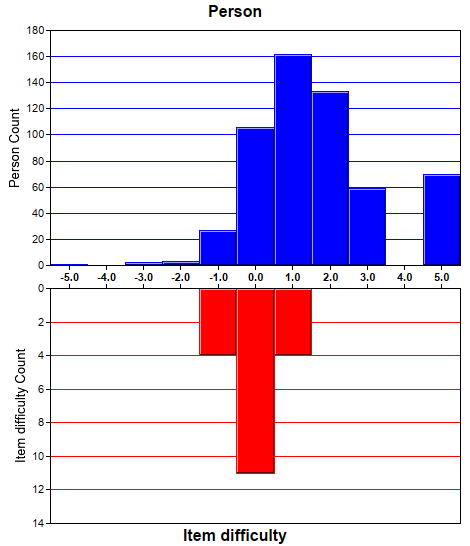


*Figure 1.* Wright map illustrating person ability in blue, and item difficulty in red for the Functional subscale of the MED-fLAG

The Wright map for the Interactive Medication Literacy subscale showed a much wider spread of participants’ ability levels in relation to item difficulty, with most of participants having interactive ability levels ranging from -6 to -2 logits and a proportion of participants reaching as high as +3 logits. This is congruent with the normalized mean score of 77.17 (SD = 20.72), indicating generally high levels of interactive medication literacy within the sample. While the participants’ ability levels were widely distributed, the item difficulties were clustered between 0 and +1 logits, and the items were generally too easy for much of the sample. These findings point to a need for developing additional, more challenging items to capture better the full spectrum of abilities in the Functional domain and enhance measurement precision.


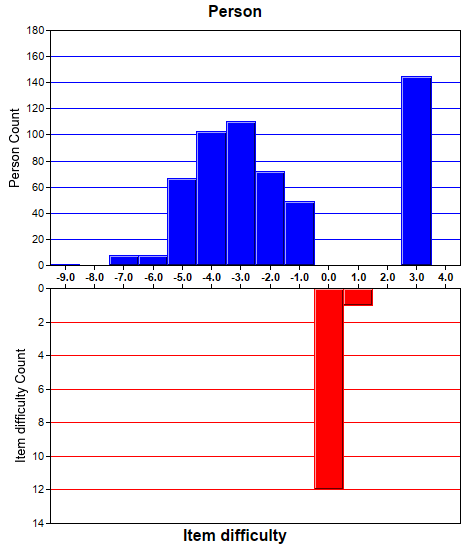


*Figure 2.* Wright map illustrating person ability in blue, and item difficulty in red for the Interactive subscale of the MED-fLAG

In contrast, the Wright map for the Critical Medication Literacy subscale revealed a notable mismatch between items’ difficulty and participants’ ability levels. While item difficulties were centered around –1 to +1 logits, most participants scored considerably lower, with abilities concentrated between –4.0 and –2.0 logits. This misalignment suggests that items in the critical subscale may be too challenging for the sample. The lower normalized mean score of 52.43 (SD = 14.75) further supports this interpretation. In addition to this misalignment, the narrow distribution of item difficulties indicates limited coverage across the spectrum of ability levels, further contributing to measurement inefficiency.


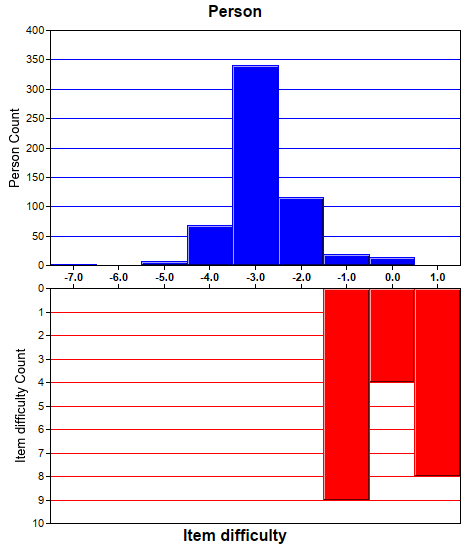


*Figure 3.* Wright map illustrating person ability in blue, and item difficulty in red for the Critical subscale of the MED-fLAG

**Appendix D**

**Differential Item Functioning (DIF) for the Functional, Interactive, and Critical medication literacy subscales, across language groups**

DIF was examined using the Rasch-Welch t-test. Following recommended Rasch criteria (Bond & Fox, 2013), statistically significant DIF was defined as |t| > 2, while practical significance was evaluated using DIF contrasts, with values ≥ 0.5 logits indicating meaningful DIF.

For the Functional Medication Literacy (FML) subscale, DIF analyses showed that most items functioned equivalently across language groups. One item (FML10) demonstrated moderate DIF (DIF contrast = 0.82 logits), indicating a meaningful difference in item difficulty between language groups. Another item (FML1) showed statistically significant DIF but with a small contrast (0.30 logits), suggesting negligible practical impact according to recommended Rasch criteria (Bond & Fox, 2013). The remaining items showed negligible DIF.

**Table D1**

**Differential item functioning (DIF) statistics for Functional Medication Literacy items showing cross-language differences**

| **Item** | **Rasch–Welch t-test** | **p-value** | **DIF contrast (logits)** | **DIF magnitude** | **Interpretation** |
| --- | --- | --- | --- | --- | --- |
| *FML1* | -2.33 | 0.020 | -0.30 | Negligible | Statistically significant but small DIF |
| *FML10* | -4.25 | <0.001 | -0.82 | Moderate | Meaningful DIF between language groups |

These items, assessing perceived difficulty (3 = not difficult at all to 0 = very difficult/impossible), were the following (free translation from French):

- FML1...*list the names of all the medicines you manage, both prescription and non-prescription (either by heart or with the help of a support such as a prescription list)*
- FML10 *...say whether the list of current medicines includes any PRN medicines (i.e. to be taken only when needed)*

*Figure 1.* DIF plot for the Functional subscale, across language groups (French-speaking vs Dutch-speaking participants)

In the Interactive Medication Literacy (IML) subscale, two items (IML28 and IML30) out of 13 demonstrated both statistically significant and practically meaningful DIF according to Rasch criteria (DIF contrasts = 0.57 and 0.87 logits, respectively). Two additional items (IML25 and IML33) showed statistically significant DIF but with contrasts below the recommended threshold of 0.5 logits (DIF contrasts = 0.43 and 0.45), indicating negligible practical impact.

**Table D2**

**Differential item functioning (DIF) statistics for Interactive Medication Literacy items showing cross-language differences**

| **Item** | **Rasch–Welch t-test** | **p-value** | **DIF contrast (logits)** | **DIF magnitude** | **Interpretation** |
| --- | --- | --- | --- | --- | --- |
| *IML25* | 2.50 | 0.013 | 0.43 | Negligible | Statistically significant but small DIF |
| *IML28* | -3.46 | <0.001 | -0.57 | Moderate | Meaningful DIF between language groups |
| *IML30* | 5.12 | <0.001 | 0.87 | Moderate | Meaningful DIF between language groups |
| *IML33* | -2.18 | 0.030 | -0.45 | Negligible | Statistically significant but small DIF |

These items, assessing perceived difficulty (3 = not difficult at all to 0 = very difficult/impossible), were the following:

- IML28 *...express your interest in natural remedies such as herbal remedies, homeopathy, food supplements*
- IML30 *...give information about known allergies or poorly tolerated drugs (including contrast media, iodine, etc.)*
- IML25 *...understand information about medicines given by health professionals*
- IML33 *...tell a health professional if you have stopped or changed the dosage of a medicine, or if you are thinking of doing so*

*Figure 2.* DIF plot for the Interactive subscale, across language groups (French-speaking vs Dutch-speaking participants)

The Critical dimension (CML) displayed the most pronounced DIF, with noticeable divergence in the curves across several items and ability levels. This pattern was consistent with the statistical identification of multiple items exhibiting cross-language DIF in the Critical subscale. In total, 14 items out of 21 showed statistically significant DIF according to the Rasch–Welch t-test, while six items (CML40, CML49, CML50, CML51, CML55, and CML56) exceeded the recommended threshold of 0.5 logits, indicating practically meaningful DIF.

**Table D3**

**Differential item functioning (DIF) statistics for Critical Medication Literacy items showing cross-language differences**

| **Item** | **Rasch–Welch t-test** | **p-value** | **DIF contrast (logits)** | **DIF magnitude** | **Interpretation** |
| --- | --- | --- | --- | --- | --- |
| *CML40* | 5.73 | <0.001 | 0.50 | Moderate | Meaningful DIF between language groups |
| *CML41* | 3.61 | <0.001 | 0.31 | Negligible | Statistically significant but small DIF |
| *CML42* | 2.73 | 0.007 | 0.23 | Negligible | Statistically significant but small DIF |
| *CML43* | 3.19 | 0.002 | 0.27 | Negligible | Statistically significant but small DIF |
| *CML46* | 4.04 | <0.001 | 0.34 | Negligible | Statistically significant but small DIF |
| *CML47* | 5.28 | <0.001 | 0.46 | Negligible | Statistically significant but small DIF |
| *CML48* | -4.00 | <0.001 | -0.45 | Negligible | Statistically significant but small DIF |
| *CML49* | -5.14 | <0.001 | -0.55 | Moderate | Meaningful DIF between language groups |
| *CML50* | -4.28 | <0.001 | -0.51 | Moderate | Meaningful DIF between language groups |
| *CML51* | -4.80 | <0.001 | -0.52 | Moderate | Meaningful DIF between language groups |
| *CML52* | -3.42 | <0.001 | -0.43 | Negligible | Statistically significant but small DIF |
| *CML54* | -3.38 | <0.001 | -0.42 | Negligible | Statistically significant but small DIF |
| *CML55* | -4.42 | <0.001 | -0.54 | Moderate | Meaningful DIF between language groups |
| *CML56* | -4.59 | <0.001 | -0.53 | Moderate | Meaningful DIF between language groups |

A selection of items is presented here. Most items showing cross-linguistic differences evaluated the frequency of actions (3 = always to 0 = never):

- [CML40] *...question the reliability of information about medicines that you find in the media, advertisements, health magazines or social networks*
- [CML41] *…carry a record of the medicines you manage (in your wallet, on your phone)*
- [CML42] *...use a treatment plan that describes the medicines that need to be taken*
- [CML44] *...use a pillbox that you prepare yourself for several days, without the help of a health professional*
- [CML47] *...keep a record of the decisions that have been made concerning the medicines you manage (new medicines initiated, those removed, changes in dosage)*
- [CML48] *...go to the same pharmacy to get medicines (having the medication file in a same and unique pharmacy)*

Other items showing cross-linguistic differences evaluated perceived difficulty (3 = not difficult at all to 0 = very difficult/impossible):

- [CML50] *...know when to schedule a prescription renewal by the physician or other health professionals*
- [CML51] *...organize the stock of medicines and know when to go to the pharmacy to get a refill or to get new medicines after prescription change*
- [CML52] *...adapt your daily routines after the list of medicines has changed (e.g. after hospitalization)*
- [CML54] *...get help from your family or people around you if you have difficulties with medicines*
- [CML56] *...know which healthcare professional to contact if you have problems with the medicines you manage*

*Figure 3.* DIF plot for the Critical subscale, across language groups (French-speaking vs Dutch-speaking participants)
